# 2-(2-Methyl-5-nitro-1*H*-imidazol-1-yl)ethyl 2-nitro­benzoate

**DOI:** 10.1107/S1600536810004186

**Published:** 2010-02-06

**Authors:** Sher Bahadar Khan, Itrat Anis, Kuldip Singh, Muhammad Raza Shah

**Affiliations:** a Department of Chemical Engineering, Yonsei University, 134, Shincheon-dong, Seodaemun-gu, Seoul 120-749, Republic of Korea; b Department of Chemistry, University of Karachi, Karachi 75270, Pakistan; c Department of Chemistry, University of Leicester, George Porter Building, University Road, Leicester LE1 7RH, England; d H.E.J. Research Institute of Chemistry, International Center for Chemical and Biological Sciences, University of Karachi, Karachi 75270, Pakistan

## Abstract

In the title compound, C_13_H_12_N_4_O_6_, the mean plane through the nitro­benzene forms a dihedral angle of 37.38 (15)° with the plane through the imidazole ring. The crystal packing is stabilized by weak inter­molecular C—H⋯O and C—H⋯N inter­actions together with π–π stacking inter­actions between nitro­benzene rings [centroid–centroid distance = 3.788 (3) Å] and between imidazole rings [centroid–centroid distance = 3.590 (2) Å].

## Related literature

For the pharmacological uses of metronidazole, see: Mao *et al.* (2009 ▶); Cosar *et al.* (1966 ▶); Bowden & Izadi (1997 ▶). For a related structure, see: Bahadur *et al.* (2009 ▶). For additional structural analysis, see: Spek (2009 ▶).
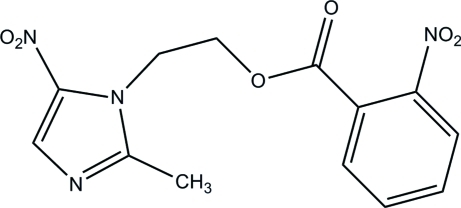


## Experimental

### 

#### Crystal data

C_13_H_12_N_4_O_6_
*M* *_r_* = 320.27
Monoclinic, 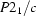

*a* = 15.392 (8) Å
*b* = 8.605 (4) Å
*c* = 10.968 (5) Å
β = 106.576 (9)°
*V* = 1392.3 (12) Å^3^
*Z* = 4
Mo *K*α radiation
μ = 0.12 mm^−1^
*T* = 150 K
0.27 × 0.24 × 0.08 mm

#### Data collection

Bruker APEX 2000 CCD area-detector diffractometer
Absorption correction: multi-scan (*SADABS*; Sheldrick, 1997 ▶) *T*_min_ = 0.573, *T*_max_ = 0.969
9743 measured reflections
2444 independent reflections
1700 reflections with *I* > \2s(*I*)
*R*_int_ = 0.099

#### Refinement

*R*[*F* ^2^ > 2σ(*F* ^2^)] = 0.056
*wR*(*F* ^2^) = 0.129
*S* = 1.00
2444 reflections
209 parameters
H-atom parameters constrained
Δρ_max_ = 0.23 e Å^−3^
Δρ_min_ = −0.23 e Å^−3^

### 

Data collection: *SMART* (Bruker, 2001 ▶); cell refinement: *SAINT* (Bruker, 2001 ▶); data reduction: *SHELXTL* (Sheldrick, 2008 ▶); program(s) used to solve structure: *SHELXS97* (Sheldrick, 2008 ▶); program(s) used to refine structure: *SHELXL97* (Sheldrick, 2008 ▶); molecular graphics: *SHELXTL*; software used to prepare material for publication: *SHELXTL*.

## Supplementary Material

Crystal structure: contains datablocks I, global. DOI: 10.1107/S1600536810004186/tk2618sup1.cif

Structure factors: contains datablocks I. DOI: 10.1107/S1600536810004186/tk2618Isup2.hkl

Additional supplementary materials:  crystallographic information; 3D view; checkCIF report

## Figures and Tables

**Table 1:** Hydrogen-bond geometry (Å, °)

*D*—H⋯*A*	*D*—H	H⋯*A*	*D*⋯*A*	*D*—H⋯*A*
C11—H11⋯O5^i^	0.95	2.58	3.474 (4)	157
C9—H9*B*⋯O6^ii^	0.99	2.45	3.166 (3)	129
C9—H9*B*⋯O5	0.99	2.39	2.838 (3)	107
C9—H9*A*⋯N2^iii^	0.99	2.57	3.513 (4)	159
C7—H7⋯O3^iv^	0.95	2.43	3.190 (4)	137

Symmetry codes: (i) 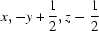; (ii) 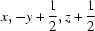; (iii) 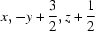; (iv) 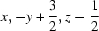.

**Fig. 1.**
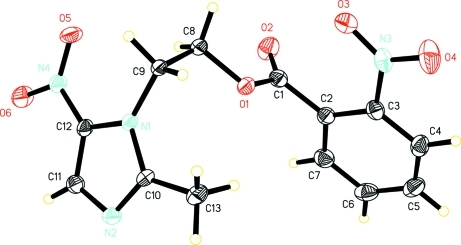
Molecular Structure of (I) with atom labelling scheme and 30% probability displacement ellipsoids.

## supplementary crystallographic information

### Comment

Metronidazole,1-(2-hydroxyethyl)-2-methyl-5-nitroimidazole, is an antibiotic which is effective against anaerobic bacteria and certain parasites (Mao *et al.*, 2009). However, there are problems related to its low aqueous solubility, toxicity and poor absorption characteristics (Bowden & Izadi 1997). In order to improve the water-solubility of metronidazole, certain esters and hemi-esters of metronidazole have been prepared (Cosar *et al.*, 1966) which have shown better solubility in aqueous medium compared to the parent metronidazole. Here, we are reporting the synthesis and crystal structure of an ester derivative of metronidazole, (I).

The molecule of (I), Fig. 1, is non-planar with a dihedral angle of 37.38 (15) ° formed between the mean planes through the nitrobenzene and imidazole rings (Spek, 2009). The nitro group is co-planar with the imidazole ring to which it is connected [dihedral angle 0.90 (3) °] , while the phenyl-nitro group is slightly twisted out of the plane of the benzene ring, forming a dihedral angle of 8.13 (3) °. The key C═O and C—N bond distances are in agreement with those observed in the related structure of 2-(2-methyl-5-nitro-1 *H*-imidazol-1-yl) ethyl 3-bromobenzoate (Bahadur *et al.*, 2009). The crystal packing is stabilized by weak intermolecular C—H···O and C—H···N interactions, Table 1 , together with π-π stacking interactions with the shortest of these occuring between symmetry related imidazole rings [ring centroid (N1–C12)··· ring centroid (N1–C12)^i^ =3.590 (2) Å for *ii*: 1-*x*, -*y*, 1-*z*]. In addition, π···π contacts are noted between symmetry related nitrobenzene rings [ring centroid (C2–C7)···ring centroid (C2–C7)^ii^ = 3.788 (3) Å for *ii* : 1-*x*, 2-*y*, 1-*z*].

### Experimental

Metronidazole (5 g, 29.23 mmol) was added to 4-nitrobenzoic acid (9.38 g, 56.11 mmol) dissolved in anhydrous CH_2_Cl_2_ (10 ml). Then, 4-dimethylaminopyridine (0.15 equiv.) and dicyclohexylcarbodiimide (1.25 equiv) were added, and the resulting solution stirred. After 12 h, the solvent was evaporated under reduced pressure. The crude reaction mixture was subjected to flash column chromatography over silica gel, successively eluting with n-hexane–ethyl acetate (2.8: 7.2) which afforded (I) in 73 % yield. Colorless crystals were obtained from the slow evaporation of a CH_2_Cl_2_ solution of (I).

### Refinement

The C-bound H atoms were geometrically placed (C–H = 0.95–0.99 Å) and refined as riding with U_iso_(H) = 1.2–1.5U_eq_(C).

### Crystal data

**Table:**

C_13_H_12_N_4_O_6_	*F*(000) = 664
*M**_r_* = 320.27	*D*_x_ = 1.528 Mg m^−^^3^
Monoclinic, *P*2_1_/*c*	Mo *K*α radiation, λ = 0.71073 Å
Hall symbol: -P 2ybc	Cell parameters from 721 reflections
*a* = 15.392 (8) Å	θ = 2.8–23.3°
*b* = 8.605 (4) Å	µ = 0.12 mm^−^^1^
*c* = 10.968 (5) Å	*T* = 150 K
β = 106.576 (9)°	Plate, yellow
*V* = 1392.3 (12) Å^3^	0.27 × 0.24 × 0.08 mm
*Z* = 4	

### Data collection

**Table:**

Bruker APEX 2000 CCD area-detector diffractometer	2444 independent reflections
Radiation source: fine-focus sealed tube	1700 reflections with *I* > \2s(*I*)
graphite	*R*_int_ = 0.099
φ and ω scans	θ_max_ = 25.0°, θ_min_ = 1.4°
Absorption correction: multi-scan (*SADABS*; Sheldrick, 1997)	*h* = −18→18
*T*_min_ = 0.573, *T*_max_ = 0.969	*k* = −10→10
9743 measured reflections	*l* = −13→12

### Refinement

**Table:**

Refinement on *F*^2^	Primary atom site location: structure-invariant direct methods
Least-squares matrix: full	Secondary atom site location: difference Fourier map
*R*[*F*^2^ > 2σ(*F*^2^)] = 0.056	Hydrogen site location: inferred from neighbouring sites
*wR*(*F*^2^) = 0.129	H-atom parameters constrained
*S* = 1.00	*w* = 1/[σ^2^(*F*_o_^2^) + (0.0572*P*)^2^] where *P* = (*F*_o_^2^ + 2*F*_c_^2^)/3
2444 reflections	(Δ/σ)_max_ < 0.001
209 parameters	Δρ_max_ = 0.23 e Å^−^^3^
0 restraints	Δρ_min_ = −0.23 e Å^−^^3^

### Special details

**Table:**

Geometry. All esds (except the esd in the dihedral angle between two l.s. planes) are estimated using the full covariance matrix. The cell esds are taken into account individually in the estimation of esds in distances, angles and torsion angles; correlations between esds in cell parameters are only used when they are defined by crystal symmetry. An approximate (isotropic) treatment of cell esds is used for estimating esds involving l.s. planes.
Refinement. Refinement of F^2^ against ALL reflections. The weighted R-factor wR and goodness of fit S are based on F^2^, conventional R-factors R are based on F, with F set to zero for negative F^2^. The threshold expression of F^2^ > 2sigma(F^2^) is used only for calculating R-factors(gt) etc. and is not relevant to the choice of reflections for refinement. R-factors based on F^2^ are statistically about twice as large as those based on F, and R- factors based on ALL data will be even larger.

### Fractional atomic coordinates and isotropic or equivalent isotropic displacement parameters (Å^2^)

**Table:**

	*x*	*y*	*z*	*U*_iso_*/*U*_eq_	
O1	0.23596 (12)	0.70542 (19)	0.54231 (16)	0.0328 (5)	
O2	0.09150 (14)	0.6281 (2)	0.5020 (2)	0.0531 (6)	
O3	0.17653 (15)	0.8476 (3)	0.73301 (19)	0.0554 (6)	
O4	0.17995 (15)	1.0918 (3)	0.7714 (2)	0.0678 (7)	
O5	0.33567 (14)	0.2348 (2)	0.51061 (18)	0.0456 (6)	
O6	0.35085 (16)	0.1756 (2)	0.3260 (2)	0.0535 (6)	
N1	0.38462 (14)	0.5437 (2)	0.47700 (18)	0.0274 (5)	
N2	0.42041 (15)	0.6345 (3)	0.3073 (2)	0.0349 (6)	
N3	0.16552 (16)	0.9819 (3)	0.6976 (2)	0.0429 (6)	
N4	0.35352 (16)	0.2691 (3)	0.4118 (2)	0.0359 (6)	
C1	0.14715 (19)	0.7286 (3)	0.5095 (2)	0.0335 (6)	
C2	0.12371 (17)	0.8940 (3)	0.4747 (3)	0.0336 (7)	
C3	0.13293 (17)	1.0152 (3)	0.5613 (3)	0.0342 (7)	
C4	0.10931 (18)	1.1655 (3)	0.5259 (3)	0.0419 (8)	
H4	0.1170	1.2449	0.5882	0.050*	
C5	0.0745 (2)	1.1999 (4)	0.3993 (3)	0.0490 (8)	
H5	0.0576	1.3035	0.3732	0.059*	
C6	0.0642 (2)	1.0840 (4)	0.3104 (3)	0.0500 (8)	
H6	0.0395	1.1076	0.2227	0.060*	
C7	0.08938 (19)	0.9331 (4)	0.3477 (3)	0.0434 (7)	
H7	0.0830	0.8546	0.2848	0.052*	
C8	0.26654 (18)	0.5514 (3)	0.5882 (2)	0.0327 (6)	
H8A	0.2339	0.4717	0.5272	0.039*	
H8B	0.2552	0.5323	0.6713	0.039*	
C9	0.36572 (17)	0.5446 (3)	0.6016 (2)	0.0287 (6)	
H9A	0.3959	0.6354	0.6514	0.034*	
H9B	0.3915	0.4495	0.6492	0.034*	
C10	0.40945 (17)	0.6699 (3)	0.4201 (2)	0.0306 (6)	
C11	0.40087 (18)	0.4813 (3)	0.2895 (2)	0.0351 (7)	
H11	0.4019	0.4231	0.2163	0.042*	
C12	0.37939 (18)	0.4234 (3)	0.3933 (2)	0.0299 (6)	
C13	0.4210 (2)	0.8282 (3)	0.4750 (3)	0.0405 (7)	
H13A	0.4510	0.8942	0.4264	0.061*	
H13B	0.4582	0.8232	0.5638	0.061*	
H13C	0.3615	0.8718	0.4712	0.061*	

### Atomic displacement parameters (Å^2^)

**Table:**

	*U*^11^	*U*^22^	*U*^33^	*U*^12^	*U*^13^	*U*^23^
O1	0.0350 (11)	0.0268 (10)	0.0376 (11)	0.0009 (8)	0.0123 (9)	0.0037 (8)
O2	0.0401 (12)	0.0407 (13)	0.0799 (16)	−0.0053 (10)	0.0196 (11)	0.0035 (11)
O3	0.0745 (16)	0.0549 (16)	0.0435 (14)	0.0126 (12)	0.0276 (12)	0.0088 (11)
O4	0.0684 (17)	0.0673 (17)	0.0586 (15)	0.0159 (13)	0.0036 (13)	−0.0246 (13)
O5	0.0764 (16)	0.0276 (11)	0.0378 (12)	−0.0041 (10)	0.0242 (11)	0.0047 (9)
O6	0.0910 (18)	0.0274 (11)	0.0468 (13)	−0.0035 (11)	0.0272 (12)	−0.0119 (10)
N1	0.0364 (13)	0.0205 (12)	0.0267 (12)	0.0006 (9)	0.0109 (10)	0.0008 (9)
N2	0.0456 (14)	0.0312 (14)	0.0310 (13)	0.0002 (11)	0.0159 (11)	0.0028 (10)
N3	0.0360 (14)	0.0465 (17)	0.0478 (17)	0.0069 (12)	0.0143 (12)	−0.0085 (14)
N4	0.0512 (15)	0.0251 (13)	0.0329 (14)	0.0018 (11)	0.0144 (12)	−0.0017 (11)
C1	0.0320 (16)	0.0359 (17)	0.0340 (16)	−0.0031 (13)	0.0115 (13)	−0.0009 (13)
C2	0.0281 (15)	0.0340 (16)	0.0417 (17)	−0.0003 (12)	0.0147 (13)	0.0030 (13)
C3	0.0250 (14)	0.0397 (18)	0.0394 (17)	0.0017 (12)	0.0115 (12)	−0.0014 (13)
C4	0.0341 (17)	0.0337 (17)	0.061 (2)	0.0015 (13)	0.0191 (15)	−0.0012 (15)
C5	0.0392 (18)	0.0387 (19)	0.072 (2)	0.0058 (14)	0.0208 (17)	0.0174 (18)
C6	0.0432 (19)	0.059 (2)	0.0473 (19)	0.0058 (16)	0.0127 (15)	0.0210 (18)
C7	0.0394 (18)	0.0483 (19)	0.0446 (19)	0.0006 (15)	0.0155 (15)	0.0031 (15)
C8	0.0459 (17)	0.0227 (14)	0.0334 (15)	0.0011 (12)	0.0177 (13)	0.0017 (11)
C9	0.0422 (16)	0.0215 (14)	0.0247 (14)	0.0032 (12)	0.0134 (12)	0.0015 (11)
C10	0.0365 (16)	0.0220 (15)	0.0345 (16)	0.0023 (12)	0.0122 (12)	0.0060 (12)
C11	0.0459 (18)	0.0310 (17)	0.0311 (16)	0.0018 (13)	0.0151 (13)	−0.0024 (12)
C12	0.0425 (17)	0.0200 (14)	0.0285 (14)	0.0023 (12)	0.0120 (12)	−0.0020 (11)
C13	0.0504 (19)	0.0251 (16)	0.0480 (18)	−0.0049 (13)	0.0172 (15)	0.0009 (13)

### Geometric parameters (Å, °)

**Table:**

O1—C1	1.326 (3)	C4—C5	1.370 (4)
O1—C8	1.448 (3)	C4—H4	0.9500
O2—C1	1.204 (3)	C5—C6	1.371 (4)
O3—N3	1.216 (3)	C5—H5	0.9500
O4—N3	1.223 (3)	C6—C7	1.384 (4)
O5—N4	1.227 (3)	C6—H6	0.9500
O6—N4	1.230 (3)	C7—H7	0.9500
N1—C10	1.360 (3)	C8—C9	1.492 (4)
N1—C12	1.371 (3)	C8—H8A	0.9900
N1—C9	1.476 (3)	C8—H8B	0.9900
N2—C10	1.331 (3)	C9—H9A	0.9900
N2—C11	1.354 (3)	C9—H9B	0.9900
N3—C3	1.462 (4)	C10—C13	1.479 (4)
N4—C12	1.417 (3)	C11—C12	1.367 (3)
C1—C2	1.491 (4)	C11—H11	0.9500
C2—C7	1.383 (4)	C13—H13A	0.9800
C2—C3	1.390 (4)	C13—H13B	0.9800
C3—C4	1.369 (4)	C13—H13C	0.9800
			
C1—O1—C8	116.1 (2)	C2—C7—H7	119.3
C10—N1—C12	105.4 (2)	C6—C7—H7	119.3
C10—N1—C9	125.2 (2)	O1—C8—C9	107.0 (2)
C12—N1—C9	129.4 (2)	O1—C8—H8A	110.3
C10—N2—C11	106.0 (2)	C9—C8—H8A	110.3
O3—N3—O4	122.8 (3)	O1—C8—H8B	110.3
O3—N3—C3	119.2 (2)	C9—C8—H8B	110.3
O4—N3—C3	118.0 (3)	H8A—C8—H8B	108.6
O5—N4—O6	123.5 (2)	N1—C9—C8	112.0 (2)
O5—N4—C12	119.6 (2)	N1—C9—H9A	109.2
O6—N4—C12	116.9 (2)	C8—C9—H9A	109.2
O2—C1—O1	124.7 (3)	N1—C9—H9B	109.2
O2—C1—C2	123.6 (3)	C8—C9—H9B	109.2
O1—C1—C2	111.6 (2)	H9A—C9—H9B	107.9
C7—C2—C3	116.2 (3)	N2—C10—N1	111.7 (2)
C7—C2—C1	119.0 (3)	N2—C10—C13	123.8 (2)
C3—C2—C1	124.8 (3)	N1—C10—C13	124.4 (2)
C4—C3—C2	123.2 (3)	N2—C11—C12	109.3 (2)
C4—C3—N3	117.5 (3)	N2—C11—H11	125.3
C2—C3—N3	119.3 (2)	C12—C11—H11	125.3
C3—C4—C5	119.2 (3)	C11—C12—N1	107.5 (2)
C3—C4—H4	120.4	C11—C12—N4	127.2 (2)
C5—C4—H4	120.4	N1—C12—N4	125.2 (2)
C4—C5—C6	119.7 (3)	C10—C13—H13A	109.5
C4—C5—H5	120.1	C10—C13—H13B	109.5
C6—C5—H5	120.1	H13A—C13—H13B	109.5
C5—C6—C7	120.4 (3)	C10—C13—H13C	109.5
C5—C6—H6	119.8	H13A—C13—H13C	109.5
C7—C6—H6	119.8	H13B—C13—H13C	109.5
C2—C7—C6	121.3 (3)		
			
C8—O1—C1—O2	−8.7 (4)	C1—O1—C8—C9	172.1 (2)
C8—O1—C1—C2	175.0 (2)	C10—N1—C9—C8	99.1 (3)
O2—C1—C2—C7	−72.3 (4)	C12—N1—C9—C8	−79.0 (3)
O1—C1—C2—C7	104.2 (3)	O1—C8—C9—N1	−70.4 (3)
O2—C1—C2—C3	107.2 (3)	C11—N2—C10—N1	0.9 (3)
O1—C1—C2—C3	−76.4 (3)	C11—N2—C10—C13	−177.9 (3)
C7—C2—C3—C4	0.5 (4)	C12—N1—C10—N2	−0.4 (3)
C1—C2—C3—C4	−178.9 (3)	C9—N1—C10—N2	−178.9 (2)
C7—C2—C3—N3	177.7 (2)	C12—N1—C10—C13	178.3 (3)
C1—C2—C3—N3	−1.7 (4)	C9—N1—C10—C13	−0.2 (4)
O3—N3—C3—C4	171.1 (3)	C10—N2—C11—C12	−1.0 (3)
O4—N3—C3—C4	−8.4 (4)	N2—C11—C12—N1	0.7 (3)
O3—N3—C3—C2	−6.3 (4)	N2—C11—C12—N4	178.9 (3)
O4—N3—C3—C2	174.2 (3)	C10—N1—C12—C11	−0.2 (3)
C2—C3—C4—C5	0.3 (4)	C9—N1—C12—C11	178.2 (2)
N3—C3—C4—C5	−177.0 (2)	C10—N1—C12—N4	−178.4 (2)
C3—C4—C5—C6	−0.2 (4)	C9—N1—C12—N4	0.0 (4)
C4—C5—C6—C7	−0.5 (4)	O5—N4—C12—C11	−179.6 (3)
C3—C2—C7—C6	−1.3 (4)	O6—N4—C12—C11	0.8 (4)
C1—C2—C7—C6	178.2 (3)	O5—N4—C12—N1	−1.7 (4)
C5—C6—C7—C2	1.4 (4)	O6—N4—C12—N1	178.8 (3)

### Hydrogen-bond geometry (Å, °)

**Table:**

*D*—H···*A*	*D*—H	H···*A*	*D*···*A*	*D*—H···*A*
C11—H11···O5^i^	0.95	2.58	3.474 (4)	157
C9—H9B···O6^ii^	0.99	2.45	3.166 (3)	129
C9—H9B···O5	0.99	2.39	2.838 (3)	107
C9—H9A···N2^iii^	0.99	2.57	3.513 (4)	159
C7—H7···O3^iv^	0.95	2.43	3.190 (4)	137

Symmetry codes: (i) *x*, −*y*+1/2, *z*−1/2; (ii) *x*, −*y*+1/2, *z*+1/2; (iii) *x*, −*y*+3/2, *z*+1/2; (iv) *x*, −*y*+3/2, *z*−1/2.
